# Experiences of discrimination and snacking behavior in Black and Latinx children

**DOI:** 10.1111/cdev.14191

**Published:** 2024-10-29

**Authors:** Katherine B. Ehrlich, Julie M. Brisson, Elizabeth R. Wiggins, Sarah M. Lyle, Manuela Celia‐Sanchez, Daisy Gallegos, Anna Langer, Kharah M. Ross, Mary A. Gerend

**Affiliations:** ^1^ Department of Psychology University of Georgia Athens Georgia USA; ^2^ Center for Family Research University of Georgia Athens Georgia USA; ^3^ Psychology Discipline Eckerd College St. Petersburg Florida USA; ^4^ Centre for Social Sciences Athabasca University Athabasca Alberta Canada; ^5^ Psychology Department University of Calgary Calgary Alberta Canada; ^6^ Department of Behavioral Sciences and Social Medicine Florida State University Tallahassee Florida USA

## Abstract

Little is known about how discrimination contributes to health behaviors in childhood. We examined the association between children's exposure to discrimination and their snacking behavior in a sample of youth of color (*N* = 164, *M*_age_ = 11.5 years, 49% female, 60% Black, 40% Hispanic/Latinx). We also explored whether children's body mass index (BMI) or sleepiness moderated the association between discrimination and calorie consumption. The significant link between discrimination and calorie consumption was moderated by children's BMI, such that discrimination was associated with calorie consumption for children with BMI percentiles above 79%. Children's sleepiness did not serve as an additional moderator. Efforts to promote health should consider children's broader socio‐contextual experiences, including discrimination, as factors that may shape eating patterns.

**Abbreviations**
BMI: body mass index
SES: socioeconomic status

Exposure to discrimination, including mistreatment due to race, age, sex, religion, weight, immigration status, or other social group status, has been linked to a range of both mental and physical health problems (e.g., depression, substance misuse, hypertension, and diabetes; Agbonlahor et al., 2024; Brown, 2017; Lewis et al., 2015; Pascoe & Richman, 2009; Williams et al., 2003, 2019). For example, in a sample of adults who were asked about their earliest experiences of racial discrimination, exposure to racial discrimination in early childhood was associated with cardiovascular‐related conditions in adulthood (Cuevas et al., 2021). These associations between discrimination and health problems, however, are not confined to adulthood (Pachter & Coll, 2009). Unfair treatment (e.g., being harassed, receiving poorer service than others) and discrimination are linked to higher blood pressure (Beatty & Matthews, 2009) and higher allostatic load, a cross‐system indicator of physiological “wear and tear,” in African American adolescents (Brody et al., 2014). Accumulating evidence suggests that exposure to discrimination at least partially accounts for the persistent racial and ethnic health disparities that have been observed for decades (Clark et al., 1999; Williams et al., 1997; Williams & Mohammed, 2009).

Experiences of discrimination activate stress‐related physiologic pathways (Heard‐Garris et al., 2024; Lawrence et al., 2022), including neuroendocrine, immune, and epigenetic processes, all of which may serve to accelerate biological aging and increase risk for poor health. Additionally, evidence suggests that discrimination shapes health behaviors in childhood and adulthood, including diet, physical activity, smoking, and alcohol use (Brodish et al., 2011; Brondolo et al., 2023; Pascoe et al., 2022). For example, studies of African American adults have shown that in response to race‐related chronic stress, some individuals turn to food to cope (e.g., Hoggard et al., 2019, 2023; Salami et al., 2019; Woods‐Giscombe et al., 2021). Studies have documented links between discrimination exposure and emotional eating, eating in the absence of hunger, snacking behaviors, loss of control eating, and choosing unhealthy food options among racially diverse samples of adults (Brown et al., 2022; Goldschmidt et al., 2017; Longmire‐Avital & McQueen, 2019; Nadimpalli et al., 2017; Pascoe & Richman, 2011). These patterns have raised concerns among clinicians, given that excess calorie consumption, if sustained, could lead to weight gain (e.g., Berkey et al., 2000) and risk for future cardiometabolic diseases, including heart disease and diabetes (Eckel et al., 1998; Hu et al., 2001).

People of color are disproportionately burdened with many chronic diseases, with disparities emerging as early as childhood (Price et al., 2013). In the last two decades, researchers have made considerable progress in identifying various psychosocial and environmental stressors (including discrimination) that might predict health behaviors, such as snacking and excess calorie consumption. For example, discrimination has been linked with stress‐induced eating behavior among African American, Latinx, and Asian American individuals—an effect that did not emerge for non‐Hispanic White individuals (Kazmierski et al., 2021). The Environmental Affordances Model proposes that the motivation to engage in unhealthy coping strategies is influenced by social structures and contexts (Mezuk et al., 2013). For example, neighborhoods tend to be racially segregated, and Black and Latinx families disproportionately reside in disadvantaged neighborhoods, where access to healthy foods and safe recreational activities may be limited. As a consequence, coping strategies may reflect these restrictions and include sedentary activities and unhealthy dietary behaviors (e.g., eating high‐fat, processed foods). In line with this model, children of color may adopt unhealthy coping strategies, such as snacking, to alleviate the distress associated with discrimination. Although snacking may benefit mental health in the short‐term, this behavior may contribute to increased chronic disease risk over time.

Notably, few studies have explored the association between discrimination and eating behaviors in children of color. This gap is surprising because awareness of discrimination begins early in childhood (Brown & Bigler, 2005; Dulin‐Keita et al., 2011), and by adolescence some youth experience unfair treatment daily (English et al., 2020). Further, eating habits develop in childhood and are stable over time (e.g., Mikkilä et al., 2005), so unhealthy diets established early in life may forecast accelerated risk for obesity in adulthood (Chang et al., 2021). Additionally, children and adolescents mostly choose unhealthy snacks (i.e., prefer sweets, fatty and salty snacks, and sweetened drinks over healthier snack options like fruits and vegetables; Almoraie et al., 2021). Indeed, over a quarter of children's daily caloric intake comes from snacking (Piernas & Popkin, 2010). Rates of snacking have increased over the last four decades, with children of color being disproportionately targeted by junk food marketing (e.g., Harris et al., 2022) and increasingly consuming unhealthy snack options (Dunford & Popkin, 2018).

In the present study, we take a multi‐method approach to test the hypothesis that more frequent exposure to discrimination in children of color will be linked to unhealthy snacking behaviors observed during a laboratory visit. We also explore two possible factors that might moderate the association between exposure to discrimination and children's snack calorie consumption. First, some evidence suggests that weight status (e.g., body mass index [BMI]) shapes the extent to which various stressors are linked to eating behavior, with overweight children (Miller et al., 2019) and adults (Lemmens et al., 2011; O'Connor et al., 2008) being most likely to overeat in response to stress. This finding reflects a general consensus that there is variability in the extent to which people are likely to over‐ versus under‐eat when stressed (Araiza & Lobel, 2018). In line with past research, we predict that the association between discrimination and calorie consumption will be stronger for children with higher versus lower BMI percentiles.

Second, we consider whether sleep plays a role in shaping the extent to which children's exposure to discrimination is linked to their snack calorie consumption. Sleep is associated with children and adults' self‐regulation and impulse control (Pilcher et al., 2015; Van Cauter et al., 2007), and low levels of these capacities may contribute to unhealthy snacking choices, including the volume of consumption (Dohle et al., 2018). Poor sleep has been linked to eating behaviors and diet quality in samples of majority‐White children and adolescents (Hart et al., 2013; Widome et al., 2019) and in a racially representative sample of U.S. adults (Jansen et al., 2020). Although this evidence suggests that poor quality or insufficient sleep may have a direct impact on eating behavior, few studies have explored whether poor sleep exacerbates unhealthy eating behaviors in children of color. However, a related study examining majority‐Black youth with overweight/obesity found that shorter sleep duration moderated the association between negative affect and loss of control eating. On days following a shorter night of sleep, children's negative affect (e.g., anxiety, sadness) was more strongly related to loss of control eating compared to days when children had longer nights of sleep (Manasse et al., 2022). In the present study, we predicted that children's sleepiness would amplify the effect of discrimination on their snacking behavior.

Finally, we conducted exploratory analyses to further examine children's snacking behavior by considering the characteristics of the snacks that children choose to eat. Studies have shown that stress increases adults' desire for sweet foods, which tend to be more rewarding than other food options (Oliver et al., 2000). Further, some research demonstrates that exposure to discrimination is associated with self‐reported sweet food choices and consumption in Belgian children (Michels et al., 2015), Black college students (Pascoe & Richman, 2011), and South Asian adults (Nadimpalli et al., 2017). We tested whether discrimination was differentially linked to children's consumption of sweet or salty snacks. We further tested whether BMI percentiles or children's sleepiness moderated these associations.

## METHOD

### Participants and study design

Children from the surrounding Athens, GA, community were recruited through local advertisements, community liaisons, and snowball recruiting with the support of existing participants in the study. To be eligible, children had to be between the ages of 8–16, English‐speaking, identify as either Black or Hispanic/Latinx, and in good health, defined as (a) without a history of chronic immune‐related diseases, such as diabetes, sickle cell anemia, or rheumatoid arthritis and (b) without acute infections for 4 weeks. (These criteria reflect the broader goals of the project, which focused on immune and cardiometabolic outcomes.) Data collection took place between July 2021 and May 2022. Guardians (93% mothers, 4% fathers, and 3% others) provided written consent, and children provided written assent. The Institutional Review Board at the University of Georgia approved all study protocols. Children and their guardian each received $50 for participating in the study, and guardians received an additional $20 for transportation.

Children and their guardians completed a baseline assessment in the lab as part of a larger 1‐year longitudinal study focused on risk and resilience in youth of color. Although 167 were enrolled in the study, we determined that one child had untreated diabetes (hemoglobin A1C value of 15.7%), and two children were unable to complete the study procedures, resulting in a final analytic sample of 164 (*M*_age_ = 11.5, SD = 2.6, range: 8–16; 49% girls; 60% Black/African American; 40% Hispanic/Latinx).

### Procedures

Research assistants provided children with surveys to complete on their own, and they were available to answer any questions. For younger children who requested assistance, a research assistant read questionnaires to participants. Additionally, research assistants monitored children's snack consumption during the study visit, which lasted about 2 h on average.

### Measures

#### Discrimination

Children reported on their experiences with discrimination using the Everyday Discrimination Scale (Williams et al., 1997), which asks about the frequency of routine experiences of unfair treatment. This 9‐item scale is validated for use with children as young as 7 (Dulin‐Keita et al., 2011). Sample items include “You are treated with less respect than other people are,” “People act as if they think you are dishonest,” and “You are called names or insulted.” Responses were scored on a six‐point Likert‐type scale ranging from 1 (*almost every day*) to 6 (*never*). Scores were reversed so that higher scores represent greater frequency of experiencing discrimination. Responses were then averaged to create an overall mean discrimination score (*α* = .82, *M* [SD] = 2.28 [1.08]). The full version of this scale allows participants to select their perceived reasons for the discrimination (e.g., gender, race, age, weight). We elected not to ask these follow‐up questions, given that (a) children can experience discrimination for multiple reasons simultaneously and (b) children's understanding about their experiences of discrimination is heavily influenced by their cognitive development (Brown, 2017), and our sample included children across multiple developmental periods. Thus, our measure of discrimination reflects children's frequency of overall experiences of discrimination rather than mistreatment due to a specific identity status.

#### Sleepiness

Children completed the sleepiness subscale of the Cleveland Adolescent Sleepiness Questionnaire (Spilsbury et al., 2007), which measures how sleepy children feel during a usual week. This measure, which has been used in samples of children as young as 8 (Li et al., 2013), asks children to respond to items such as “I fall asleep during my morning classes” and “I feel drowsy if I ride in a car for longer than 5 min” using a 1 (*never*) to 5 (*almost every day*) scale. Responses were averaged to create an index of sleepiness (*α* = .83, *M* [SD] = 2.22 [0.79]).

#### BMI

Children's height was measured using a stadiometer (Seca), and weight was measured using a digital scale (Health O Meter). Weight was recorded twice to the nearest 0.1 kg; if measures differed by more than 0.2 kg, a third measure was taken. The two closest weights were averaged. Children's height was similarly measured twice. Heights were recorded to the nearest 0.1 cm and were measured a third time if measures differed by more than 0.2 cm. The two closest height measurements were then averaged. BMI was calculated using the standard formula (weight in kilograms divided by height in meters squared). Scores were then transformed to sex‐ and age‐adjusted percentiles (Centers for Disease Control and Prevention, 2023).

#### Calorie consumption

During the study visit, children were offered a variety of snacks, including chips, cookies, pretzels, crackers, and fruit drinks. A box containing a selection of pre‐packaged snacks (each with labeled calorie contents) was available to children throughout the study, and children were told that they could eat as many snacks as they wanted. Research assistants monitored children's snack consumption by recording what snacks were eaten; this information was later tallied to calculate the total calories consumed. Children almost always ate the entire contents of a snack package, but to be consistent across participants, research assistants marked a snack as completely consumed as long as a child consumed at least some portion of a snack. Children's calorie consumption ranged from 0 to 1470 calories (*M*_calories_ = 249.6, SD = 258.9). We also sorted children's snack calories based on whether the items were *sweet snacks* (e.g., cookies, fruit snacks) or *salty snacks* (e.g., pretzels, chips).

#### Covariates

We selected the following covariates based on a priori hypotheses about factors that may be related to calorie consumption. These covariates were included in all models.

##### Child demographics

Families met with research assistants at the beginning of the study visit and provided information about the child's sex at birth, age, and race/ethnicity.

##### Socioeconomic status risk

Caregivers provided information about their families' socioeconomic status (SES). Six dichotomous variables formed a socioeconomic risk index, which was based on previous research (Brody et al., 2018). A score of 1 was assigned to each of the following: poverty based on federal guidelines, primary caregiver unemployment, receipt of government assistance (e.g., Temporary Assistance for Needy Families, Supplemental Nutrition Assistance Program benefits), primary caregiver single parenthood, primary caregiver education level less than high school graduation (or general education diploma equivalent), and caregiver reported inadequacy of family income. Scores were summed to form a single risk index, which ranged from 0 to 6 (*M* = 2.57, SD = 1.39), with higher scores indicating more socioeconomic risk.

#### Data analysis and availability

Analyses were tested using multiple regression with Hayes' PROCESS macro (v4.2, Model 2; Hayes, 2022) in SPSS. Age, sex, race/ethnicity, and SES risk were included as covariates in all models. Continuous variables used to create interaction terms were centered prior to analysis (using macro settings). Data are available upon request to the lead author.

## RESULTS

Table 1 presents descriptive statistics and bivariate correlations for the sample. Of note, youths' reported discrimination experiences and sleepiness (but not BMI percentile) were positively correlated with calorie consumption. Additionally, age was negatively correlated with calorie consumption.

**TABLE 1:** Correlations and descriptive statistics.

	*M* (SD) or %	1	2	3	4	5	6	7	8	9	10
1. Sex, female	49.1%	‐	−.037	.010	−.089	.083	−.073	.130	−.021	−.122	.108
2. Age	11.5 (2.6)		‐	.010	−.026	−.162*	−.209**	.015	−.296***	−.264***	−.182*
3. Race/ethnicity, Black	60.0%			‐	.211**	.125	.091	.264***	.148^†^	.157*	.060
4. SES risk	2.57 (1.39)				‐	.189*	.116	.191*	.113	.072	.104
5. BMI percentile	80.02 (20.98)					‐	.086	.119	.127	.150^†^	.035
6. Discrimination	2.28 (1.08)						‐	.196*	.281***	.248**	.176*
7. Sleepiness	2.22 (0.79)							‐	.157*	.121	.118
8. Total calories	249.6 (258.9)								‐	.805***	.721***
9. Sweet snack calories	116.9 (182.0)									‐	.169*
10. Salty snack calories	132.7 (155.8)										‐

*Note*: *N* = 164.

Abbreviations: BMI, body mass index; SES, socioeconomic status.

^†^ *p* < .10;

* *p* < .05;

** *p* < .01;

*** *p* < .001.

### Discrimination, caloric consumption, and moderation by sleepiness and BMI

**FIGURE 1:**
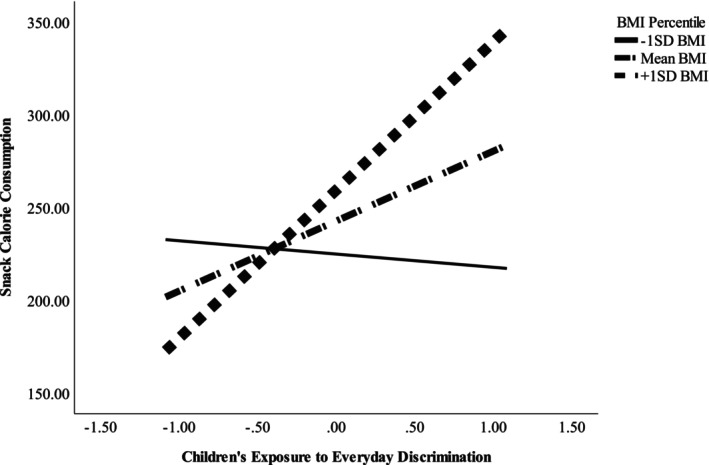
Interaction between children's exposure to discrimination (mean‐centered) and body mass index (BMI) percentile predicting children's snack calorie consumption. The figure shows estimated regression lines at ±1 SD and the mean of children's BMI percentiles.

Our primary analysis focused on whether the association between children's experiences of discrimination and calorie consumption varied as a function of their sleepiness or BMI percentile (Table 2). A significant Discrimination × BMI percentile interaction emerged (*b* = 1.94, SE = 0.90, *p* < .05; see Figure 1). To interpret this effect, we plotted the estimated levels of calorie consumption by discrimination at low (1 SD below the mean) and high (1 SD above the mean) BMI values, while also controlling for children's sleepiness (see Figure 1). These analyses revealed that, for youth with higher BMI percentiles, discrimination was positively associated with calorie consumption (conditional effect = 79.3, SE = 22.9, *p* < .001). Using the Johnson‐Neyman technique, we found that the link between discrimination and calorie consumption was significant for youth with BMI percentiles above 79%, which was 63.4% of this sample. For youth with lower BMI percentiles, however, there was no association between discrimination and calorie consumption (*p* = .81). In addition, the Discrimination × Sleepiness interaction was not significantly associated with calorie consumption (*p* = .16; Table 2).

**TABLE 2:** Discrimination and sleepiness as predictors of snack calorie consumption.

Predictors	Total calories		Sweet snack calories		Salty snack calories	
	*b*	[95% CI]	*b*	[95% CI]	*b*	[95% CI]
Sex, female	−24.19	[−99.34, 50.97]	−60.37*	[−113.11, −7.63]	36.19	[−12.25, 84.62]
Age	−28.64***	[−43.64, −13.64]	−18.89***	[−29.42, −8.37]	−9.75*	[−19.41, −0.08]
Race/ethnicity, Black	54.94	[−23.71, 133.59]	46.86^†^	[−8.33, 102.06]	8.07	[−42.62, 58.76]
SES risk	2.25	[−25.66, 30.15]	−6.78	[−26.36, 12.80]	9.02	[−8.96, 27.01]
BMI percentile	0.86	[−0.97, 2.70]	1.11^†^	[−0.17, 2.40]	−0.25	[−1.43, 0.93]
Discrimination	36.79*	[0.72, 72.86]	19.12	[−6.20, 44.43]	17.67	[−5.58, 40.92]
Sleepiness	31.05	[−19.27, 81.36]	20.09	[−15.23, 55.40]	10.96	[−21.47, 43.39]
Discrimination × BMI percentile	1.94*	[0.17, 3.71]	1.81**	[0.57, 3.05]	0.12	[−1.02, 1.26]
Discrimination × sleepiness	31.63	[−12.18, 75.43]	27.66^†^	[−3.08, 58.40]	3.97	[−24.26, 32.20]

*Note*: *N* = 164.

Abbreviations: BMI, body mass index; SES, socioeconomic status.

^†^ *p* < .10;

* *p* < .05;

** *p* < .01;

*** *p* < .001.

### Salty versus sweet snacks

We then examined whether the observed effect differed as a function of the types of snacks that youth consumed, including salty snacks (e.g., chips, pretzels, crackers) and sweet snacks (e.g., cookies, granola bars, fruit snacks, fruit drinks). A significant Discrimination × BMI percentile interaction emerged in the prediction of sweet snack calories (*b* = 1.81, SE = 0.63, *p* < .01; see Table 2). The pattern of this effect mirrored the original analysis: For youth with higher BMI percentiles, discrimination was positively associated with sweet calories consumed (conditional effect = 58.6, SE = 16.15, *p* < .001); for youth with lower BMI percentiles, there was no association between discrimination and sweet snack calories (conditional effect = −21.6, SE = 20.0, *p* = .28). In contrast, the Discrimination × BMI percentile interaction predicting salty snack calories was not significant (*b* = 0.12, SE = 0.58, *p* = .83). Similarly, the Discrimination × Sleepiness interaction was not significant for either sweet or salty calorie consumption.

## DISCUSSION

Our analyses revealed that BMI, but not sleepiness, moderated the association between Black and Latinx children's discrimination experiences and calorie consumption while snacking, such that frequency of discrimination was positively associated with calorie consumption for children with BMI percentiles above 79%, which constituted 63.4% of the sample. Moreover, exploratory analyses suggested that this effect was driven by children's tendencies to consume sweet snacks. These findings mirror previous literature that suggests a link between stress and self‐reported sweet food consumption (Michels et al., 2015) and extend the literature by documenting this effect with a measure of children's *actual* eating behaviors in the lab. Collectively, our research adds to the literature linking stress to children's unhealthy eating behaviors (Hill et al., 2018) by highlighting the frequency of children of color's experiences with discrimination as an additional external stressor that may contribute to their eating patterns.

In both children and adults, emotional eating has been connected to ongoing stress (Lazarevich et al., 2016; Michels et al., 2012), and people often report consuming high‐calorie, sweet, or high‐fat foods as a means to cope with negative emotions. If sustained, however, this pattern could contribute to weight gain and risk for future cardiometabolic diseases. Notably, in our sample, the association between discrimination and calorie consumption was only significant for children with elevated BMIs. This finding likely reflects variability in children's coping strategies when faced with chronic stressors, such as discrimination. When some children of color use food to cope with discrimination, one consequence may be higher body weight over time; in contrast, other children may incorporate alternative strategies when managing negative emotions or stressful situations. For example, youth may engage in physical activity, self‐care behaviors, or pleasant interactions with friends and family, which may decrease the possibility of excess calorie consumption and elevated weight. Efforts to identify other adaptive coping mechanisms may be important for youth of color in particular as they begin to establish health habits that are likely to continue into adulthood.

Unexpectedly, although children's sleepiness was positively correlated with children's calorie consumption, it did not emerge as a significant moderator of the link between discrimination and calorie consumption. Our assessment of children's sleep focused on indicators of daytime tiredness (e.g., falling asleep at school, feeling sleepy in the car). It may be that other indicators of children's sleep are more likely to amplify the detrimental effects of discrimination on their snack behavior. For example, insufficient sleep has been shown to amplify the effects of children's negative affect on self‐reported eating behaviors (Manasse et al., 2022). Some evidence suggests that insufficient sleep shapes appetitive hormones, including leptin and ghrelin (Lin et al., 2020); as such, it may be that insufficient sleep duration, rather than sleepiness, would exacerbate the effects of discrimination on children's snacking behaviors.

Although not the primary focus of our investigation, our analyses also showed that children's self‐reported discrimination was correlated with their reports of sleepiness, which is consistent with established literature on the effects of discrimination on sleep (see Slopen et al., 2016, for a review). Given the cross‐sectional nature of our study, we did not examine possible mechanisms that might help explain why discrimination was associated with calorie consumption, but one possibility is that insufficient sleep erodes children's self‐control around snacking behavior. Such models will be exciting avenues for future research.

These findings provide new insight into psychosocial factors that may affect snacking behavior in children of color. One of the strengths of our study was the use of multiple methods to assess key constructs, including behavioral observations of children's snacking behavior, self‐reports from children about their sleepiness and experiences of discrimination, and body measurements to capture BMI. Although some studies of children's eating behaviors incorporate children's actual calorie consumption during a structured task (e.g., Salvy et al., 2012; Senese et al., 2020), many studies rely on self‐reports (e.g., van Ansem et al., 2014), which is a concern because children often underreport their calorie consumption (Forrestal, 2011). A second strength is our focus on understanding associations between discrimination and snacking behavior in a sample of children. To date, research on racial health disparities has focused on adult samples, but the roots of these health disparities can often be traced back to childhood (Johnson, 2018). Our study adds to the growing body of research highlighting the ways in which experiences of discrimination are linked to health behaviors that, over time, may have implications for development of chronic disease.

Despite these strengths, several limitations will be important to address in future research. First, children's snacking tendencies observed in the laboratory may vary due to a variety of extraneous factors (e.g., time of day, prior food consumption, snack options available). Study visits occurred primarily in the afternoon (62% of visits occurred between 2 and 5 pm), and all participants were asked to refrain from eating in the two hours leading up to the visit. Nevertheless, it is still possible that our snapshot of children's calorie consumption may not reflect their typical eating patterns at home. Additionally, although children almost always consumed the entire contents of a snack package, research assistants recorded total calorie consumption of a snack even if some portion was left uneaten. This decision may have resulted in a small number of children receiving a marginally inflated estimate of their snack calorie consumption. Second, due to funding constraints, we relied on children's self‐reports of their sleepiness rather than objective measures of sleep quality (e.g., actigraphy). Finally, the cross‐sectional study design limits our ability to make conclusions about whether discrimination causes increased snack consumption. Some experimental evidence, however, suggests that mistreatment from others (e.g., in the form of social rejection or ostracism) and stigmatizing messages about weight (e.g., weight‐stigma) are causally related to increased food intake (Major et al., 2014; Salvy et al., 2011, 2012; Schvey et al., 2012; Senese et al., 2020).

One question that these findings raise is whether discrimination is linked to calories consumed during mealtimes and over the course of the day. Future studies could employ diet recall interviews, daily diary assessments, or wearable cameras (e.g., Gage et al., 2020) to better capture a more holistic assessment of food intake and risk for overweight/obesity. Still, snacking is a central part of children's diets (Piernas & Popkin, 2010), and frequent snacking is associated with an overall higher intake of calories for children (Larson & Story, 2013). Whether children's discrimination experiences are linked to calories consumed during mealtimes or over the entire course of the day remains a question for future research.

Another question that will be important to consider in future research is whether certain forms of discrimination are most likely to be associated with snacking behavior in children of color. Because of the wide age range in our study, we chose not to ask children to identify the reasons why they experienced mistreatment, as this ability is influenced by cognitive development and may be particularly difficult for younger children (Brown, 2017). Studies with adolescents and adults will be better equipped to address questions about how certain forms of discrimination may be more strongly linked to snacking behavior relative to others. Additionally, these studies could test questions about compounding effects of discrimination across the intersection of multiple social groups (e.g., race and weight, or gender and social class).

These findings suggest that experiences of discrimination among Black and Latinx children are associated with higher consumption of sweet snacks, particularly for children with higher BMI. Public health efforts to curb the rising rates of childhood obesity have focused overwhelmingly on efforts to promote healthy diets and regular physical activity (American Heart Association et al., 2006). These efforts target logical starting points for prevention and intervention, but many interventions have been shown to be ineffective (Bomberg et al., 2023). Calls to consider the contexts in which children are living point to a recognition that social factors play an important role in children's obesity (Birch & Ventura, 2009; Skinner et al., 2023), and our findings suggest that addressing psychosocial factors should be an integral component of programs that are designed to address children's eating behavior. Effective school‐based interventions to reduce discrimination in children (Brown, 2017) and efforts to support racial socialization could be promising pathways to decrease children's exposure to discrimination and improve coping strategies in the face of mistreatment. Collectively, such efforts could have a positive impact on children's mental and physical health (Anderson & Stevenson, 2019; Brown, 2017; Clendinen & Kertes, 2022).

## FUNDING INFORMATION

This research was supported by the Jacobs Foundation (Early Career Research Fellowship 2018‐1288‐07) and the Brain and Behavior Research Foundation (Young Investigator Grant #27302), and grant P50 DA051361 from the National Institutes of Health.

## CONFLICT OF INTEREST STATEMENT

The authors declare no conflicts of interest.

## Data Availability

The data, materials, and analytic code necessary to reproduce the analyses presented here are not publicly accessible. The analyses were not preregistered.
